# Deep Feature Learning for Sudden Cardiac Arrest Detection in Automated External Defibrillators

**DOI:** 10.1038/s41598-018-33424-9

**Published:** 2018-11-21

**Authors:** Minh Tuan Nguyen, Binh Van Nguyen, Kiseon Kim

**Affiliations:** School of Electrical Engineering and Computer Science, Gwangju Institute of Science and Technology, Gwangju, 61005 South Korea

## Abstract

Ventricular fibrillation and ventricular tachycardia (VF/VT), known as shockable (SH) rhythms, are the mainly cause of sudden cardiac arrests (SCA), which is cured efficiently by the automated external defibrillator (AED). The performance of the shock advice algorithm (SAA) applied in the AED has been improved by using machine learning technique and variously conventional features, recently. In this paper, we propose a novel algorithm with relatively high performance for the SCA detection on electrocardiogram (ECG) signal. The algorithm consists of a convolutional neural network as a feature extractor (CNNE) and a Boosting (BS) classifier. A grid search with nested 5-folds cross validation (CV) is used to select the CNNE trained with preprocessed ECG, SH, and NSH signals using the modified variational mode decomposition technique. The deep feature vector learned by this CNNE is extracted at the first fully connected layer and then fed into BS classifier to validate its performance using 5-folds CV procedure. The secondary learning of the BS classifier and the use of three input channels for the CNNE improve certainly the detection performance of the proposed SAA with the validated accuracy of 99.26%, sensitivity of 97.07%, and specificity of 99.44%.

## Introduction

The sudden cardiac arrest (SCA), caused by the shockable (SH) rhythm including ventricular fibrillation (VF) and ventricular tachycardia (VT), results in unexpected deaths out of clinic environment. The out-of-hospital-cardiac-arrest (OHCA) is treated effectively by the automated external defibrillator (AED), which detects rapidly the SH rhythms on electrocardiogram (ECG) and deliver an countershock to recover the normal sinus rhythms of the heart from the distorted cardiac electrical activity. Therefore, short response time for delivering defibrillation obviously improves the life-saving chances^1^.

The most important factor of the AED is the shock advice algorithm (SAA) for that the performance needs to be complied with the American Heart Association (AHA) recommendations^2^. The rationale behind the AHA recommendations for which the Sp of 95% is higher than the Se of 90% is that incorrect detection of non-shockable (NSH) rhythms results in delivering defibrillation, which may cause an artificial SCA. However, the increase in the SAA performance is still paid intensive attention to minimize the risk of incorrect diagnosis of the practical AED.

In an effort to improve the performance, most of the recent SAA designs in the literature have counted on conventional feature extraction (FE) schemes using the stand-alone electrocardiogram (ECG)^3,4^, alternative signals^5–7^ or both of them^8^ and machine learning (ML) approaches for classification^3–8^. Obviously, the performance of the classification models is significantly dependent on the quality of the extracted input feature space and the correlation between individuals within the completed combinations using both stand-alone ECG and NSH signals with modified VMD (MVMD)^8^. Therefore, the most representative and informative set of input features, which are powerful for practical SH rhythm recognition, is needed to be addressed. This is accomplished by adopting the feature selection (FS) algorithms to eliminate the irrelevant features from the input feature space and improve the learning process of the ML classifiers^9^. Nevertheless, the FS is the time consuming algorithm and complexity due to human expert knowledge based requirements. Moreover, the shockability oriented signal (SH signal) has not been investigated with respect to improvement of SH/NSH rhythm classification performance.

The convolutional neural networks (CNN) has been widespreadly applied for biomedical signal processing and application design problems. Indeed, a design of computer-aided diagnosis system using the CNN of eleven layers to detect atrial fibrillation, atrial flutter, and VF is proposed^10^. A similar structure of the CNN is also suggested for myocardial infarction diagnosis^11^. Moreover, a CNN of 13 layers using raw stand-alone ECG signal as the input for atrial fibrillation detection produces better performance than that using the conventional features^12^. In addition, a combination of the conventional features and deep features learned by the deep neural networks (DNN) using raw ECG signal is proposed as the input of the ensemble XGBOOST classifier for diagnosis of atrial fibrillation^13^. The first research adopting deep learning (DL) technique for SCA detection is proposed in which the SAA is designed as the eleven-layer deep CNN^14^. In general, the rational behind the use of the CNN is the outstanding characteristics such as strong feature learning capabilities, less complexity because of no requirement of conventional expertise-based FE and FS algorithms^14^. However, previous study^14^ only pays attention to a specific CNN structure using the stand-alone ECG signal for the SAA design, which leads possibly to omit other effective structures of the CNN. Furthermore, the CNN feature learning has not been considered properly for training an independent ML classifier in researches of SAA design to improve the performance of the SCA detection. Last but not least, it is necessary to investigate the utility of multiple input channels such as stand-alone ECG segment, SH and NSH signals other than that of stand-alone ECG segment as a single input channel for the CNN to improve the quality of the feature learning.

In this paper, a novel SAA exploiting the advantages of both DL and ML techniques is proposed to improve the detection performance of the SCA. Obviously, the SAA performance plays an vital role in minimizing unnecessary defibrillation, which results definitely in unexpected deaths, and improving life-saving opportunities, especially, for OHCA situations without the emergency service provided at very early moments. Here, the method procedures are developed to select carefully the proposed SAA design including a CNN extractor (CNNE) and a Boosting (BS) classifier. Firstly, the MVMD technique is used to reconstruct SH and NSH signals, which have most of power of SH and NSH components inside the bandwidths below and above 10 Hz, respectively, from preprocessed ECG segment (pECG)^8,15^. Three above signals are then arranged as the input channels for the CNN. Thereafter, a grid search with nested cross validation (CV) is provided to select the best structures of the CNNEs and a full CNN model (fCNN) on the entire training data based on the minimum balanced error rate (BER) of different ML classifiers and fCNN models. The selected fCNN and the learned feature (LF) sets extracted by the CNNEs, which are used as the input of various ML classifiers, are validated on the evaluation data. Our first main contribution is the use of three 1-dimension signals (pECG, SH, and NSH signals) as the input channels of the CNN using the MVMD technique to improve the quality of the LF. Secondly, different CNN structures are investigated using the grid search with nested 5 fold-CV to select carefully the best CNNE. Lastly, the use of the deep LFs for secondary training of the ML classifier definitely improves the final classification performance of the SH/NSH rhythms.

## Databases and Preprocessing

The public databases used in this work are the Creighton University Ventricular Tachyarrhythmia Database (CUDB) and the MIT-BIH Malignant Ventricular Arrhythmia Database (VFDB). There are 35 single-channel records of around 8 minutes and 22 double-channel records of 35 minutes in the CUDB and VFDB, respectively, with sample frequency of 250 Hz. A total of 57 records in the databases for which each record corresponds to an individual patient are divided into 70% of training and 30% of evaluation sets corresponding to 40 and 17 records, respectively. The databases are then divided into non-overlapping 8 s-segments including 1135 SH segments and 5185 NSH segments^8^. The construction of the independent training and evaluation data is implemented with 4303 and 2017 segments, respectively. The signal annotations in the databases are VF, VT, and ventricular flutter for SH rhythms and others for NSH rhythms. We use the first channel of the ECG records to obtain the better learning process. According to previous publications^2,8^, the artifacts, noise, asystole, transition rhythms, slow VT of intermediate rhythms with rate under 150 beat per minute, and peak-to-peak amplitude under 200 *μ*V of VF rhythms are removed from the databases because of unclear classification, which archives no defibrillation benefit.

The ECG databases are preprocessed by existing techniques^8^, which have been proven their performance in signal processing related researches. Firstly, the smooth ECG signal is generated as the output of the five-order moving average filter. Secondly, the drift suppression and the base line wander with frequencies under 1 Hz are removed by the high-pass filter. Lastly, the low-pass Butterworth filter is employed to suppress high frequency interference above 30 Hz.

## Method

The procedures of our method includes channel construction, CNN selection, and CNN validation phases. In the first phase, the ECG databases are segmented and preprocessed. Then, the MVMD technique is used to generate SH and NSH signals from pECG, which are fed into CNN as three input channels. In the second phase, a grid search with nested 5-folds CV is implemented to select the a best fCNN and three candidate CNNEs using different ML classifiers. In the last phase, a fCNN and various ML classifiers using feature vectors extracted by three selected CNNEs are validated on evaluation data using 5-folds CV procedure.

### Channel Construction

There are three input channels of the CNN, which are preprocessed ECG segment, SH and NSH signals. Here, the MVMD is used to reconstructed SH and NSH signal from preprocessed ECG segment.

The maximum spectral amplitude of VF/VT components is at 4 Hz but dropped dramatically on the frequencies above 10 Hz. Moreover the maximum amplitude of spectrum is between 4 Hz and 20 Hz for individual QRS complexes^15^. Hence, the powers of the SH and NSH rhythms can be concentrated on the bandwidth lower and above 10 Hz. We employ the MVMD algorithm^8^ to decompose the preprocessed ECG segment into 10 modes using 5 values of center frequency inside the bandwidth of [0,10] Hz, which are 2, 3.5, 5, 6.5, 8 Hz. These center frequencies are fixed during MVMD updating procedures. The remaining center frequencies are updated and assigned automatically the values above 10 Hz during updating procedures. The first mode corresponding to zero frequency is removed due to low interference. The sum of 5 modes corresponding to such 5 values of center frequency, which has most of it power inside the bandwidth lower than 10 Hz, is the SH signal. The sum of 4 remaining modes is considered as NSH signal, which has major power spectrum on the bandwidth above 10 Hz.

**Algorithm 1:**
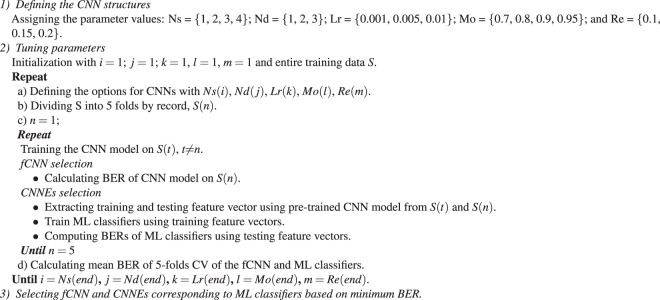
fCNN and CNNEs selection.

### CNN Selection

A grid search with nested 5-folds CV is deployed to select the best CNN structure in terms of its learning and structure parameters for the fCNN and CNNEs using the SVM, KNN, and RF classifiers on the training data. We select conventional values of these parameters, which are commonly used as shown in Algorithm 1.

Learning parameters^10,11,14^

- *Re*: This parameter prevents the network from becoming overly dependent on any one neuron called overfitting, which is common problem in neural networks.
- *Mo*: A learning property, which causes the weight change to keep going continuously in current direction. This parameter control the learning speed of the network.
- *Lr*: To determines how far each iteration of training will take the weight values to help in convergence of the data.
  Structure parameters^16,17^
- *Nd*: Two consecutive convolutional layer and ReLu are defined as a block. Nd is number of consecutive blocks, which represent the depth of the network.
- *Ns*: Each NS consists of numerous blocks, which are followed by a max pooling layer. Indeed, the use of multiple convolutional layers in a section of network improves the data representations before losing the information due to decrease in data dimensions on max pooling layer.

It is noteworthy that we use two consecutive fully connected layers (FC) for which 2 outputs of last FC represents the binary classes. Moreover, the FC determines the nonlinearity of the high level features extracted by previous layer. Therefore, the first FC is used to extract the feature vector, which is then fed into various ML classifiers.

### CNN Validation

The 5-folds CV procedure is implemented by dividing evaluation data into 5 parts by record for which 4 parts are used as training data and remaining part is for testing data. The completed process contains 5 runs to ensure that every single part is used as the testing data.

A fCNN is validated on the evaluation data using above 5-folds CV procedure. Moreover, 3 feature vectors extracted by 3 CNNEs from the evaluation data are used for the 5-folds CV procedure to evaluate the performance of 6 ML classifiers. A repetition of 100 times is applied for the 5-folds CV procedure to compute the mean and standard deviation of the performances of the fCNN and the ML classifiers. The final algorithm is selected according to highest Ac among the others of the ML classifiers and the fCNN.

## Results

We have selected fCNN^18^ for both selection and validation. Moreover, we also combine the CNN with 3 popular ML classifiers namely Support vector machine (SVM)^19^, K-nearest neigbour (KNN)^20^, and Random forest (RF)^21^ for selection of 3 CNNEs. Validation of selected CNNEs is implemented with 6 ML classifiers known as SVM, KNN, RF, Bagging (BG)^21^, BS^21^, Logistic regression (LR)^22^.

### CNN Selection

There are 432 fCNNs and 432 CNNEs corresponding to each ML classifier with different values of parameters, which are investigated on the training data using grid search with nested 5-folds CV. Table 1 shows a fCNN and 3 CNNEs, which are selected on the 8 s-segment training data using 3 ML classifiers. Here, the parameters selected for the grid search are regularization (Re), Momentum (Mo), Learning rate (Lr), Network deep (Nd), and Network section (Ns). Moreover, we use 4 main measures, which are accuracy (Ac), Se, Sp, and balanced error rate (BER) to estimate the performance of the fCNN and ML classifiers^8^.

**Table 1:** The selection of fCNN and CNNEs with their parameters on 8 s-segment training data.

fCNN and CNNEs	ML	Ns	Nd	Lr	Mo	Re
fCNN		3	1	0.005	0.7	0.1
CNNE1	SVM	1	3	0.01	0.8	0.1
CNNE2	KNN	1	2	0.02	0.9	0.2
CNNE3	RF	3	1	0.005	0.9	0.15

### CNN Validation

Table 2 presents the validation results of a fCNN and 3 CNNEs on the evaluation data. Generally, all of the ML classifiers show relative high validation performance using feature vectors extracted by the selected CNNEs on the evaluation data. The highest performance is produced by the BS classifier using the feature vector extracted from the 8 s-segment evaluation data. This feature vector is generated by the CNNE3, which is selected by the RF classifier on the training data.

**Table 2:** Highest validation performance of the fCNN and the ML classifiers using feature vectors extracted by the three CNNEs on the 8 s-segment evaluation data.

fCNN and CNNEs	ML	Ac (%)	Se (%)	Sp (%)	BER (%)
fCNN		98.36 ± 0.92	89.25 ± 4.73	99.23 ± 0.56	5.76 ± 2.46
CNNE1	BS	98.82 ± 0.51	95.52 ± 1.67	99.07 ± 0.60	2.70 ± 0.86
CNNE2	KNN	98.70 ± 0.51	95.35 ± 1.17	98.94 ± 0.57	2.85 ± 0.67
CNNE3	**BS**	**99**.**26** ± **0**.**26**	**97**.**07** ± **1**.**07**	**99**.**44** ± **0**.**29**	**1**.**74** ± **0**.**55**

### Proposed SAA for SCA Dignosis

**Figure 1:**
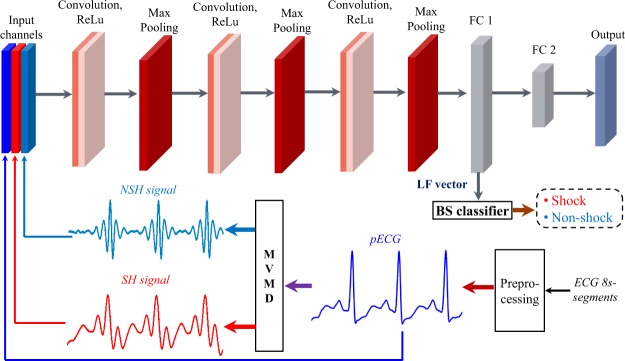
Diagram of the proposed SAA.

The proposed SAA includes a 11-layered CNNE as shown in Table 3 and BS classifier using three input channels of pECG, SH, and NSH signals generated by the MVMD technique for the 8-s segment length. The block diagram of the proposed SAA is presented in Fig. 1. Diagnosis of SCA is implemented by the SAA as follows

- A full length of 8 s-segment is collected by a slipped window in the real clinic environment and preprocessed to archive pECG.
- MVMD is applied to generate NSH and SH signals from pECG.
- pECG, NSH, and SH signals are fed into pre-trained CNNE to activate a feature vector.
- The BS classifier uses above feature vector to assign label 1 or 0 to original 8 s-segment as SH or NSH ECG segment.

**Table 3:** The selected CNNE structure details.

Layer	Filter size	Filter number	Stride	Padding
Input	2000 × 3 channels			
Convolution + ReLU	101 × 1	10	1	50
Max Pooling	11 × 1		2	
Convolution + ReLU	101 × 1	20	1	50
Max Pooling	11 × 1		2	
Convolution + ReLU	101 × 1	40	1	50
Max Pooling	11 × 1		2	
First FC	100 ouputs

A time period, which is consumed by the proposed SAA, is computed as 7.0 ± 3.5 s for 50 consecutive 8 s-segments. This interval includes segment collection, preprocessing, signal generating, feature vector activation, and classification. It is noteworthy that the time consumed by the SAA should be smaller than the segment length of 8 s to avoid the delay of diagnosis between two consecutive segments.

## Discussions

To date, the effective method for treatment of the OHCA is the portal, inexpensive, reliable electronic device namely AED. Although the relatively high Se and Sp are recommended by the AHA for the design of the SAA, the improvement of the detection performance is vital requirement to avoid erroneous diagnosis of the practical AED putting the patients into dangerous situations. Another electronic equipment, which has been used commonly for the SCA treatment, is the implantable cardioverter defibrillator (ICD). However, the Se of 100% is recommended for this device^23^, which is opposite to that of the AED. It is because the ICD delivers the discharge countershock, which carries a small amount of electrical power in comparison with defibrillation provided by the AED to treat the VF/VT rhythms. Hence, the incorrect electrical shock of the ICD causes negligible impact while that generates probably an artificial SCA for the patient if delivered by the AED.

The public CUDB and VFDB databases used as parts of training and validation for the proposed SAA are small, which may cause unstable behavior in classification performance, especially, overfitting problem^24^. Therefore, the 5-folds CV is applied for both training and validation phases to compute statistical performance results, which represent reliably the proposed algorithm performance.

The combination of pECG and its NSH signal is used for FE to investigate an expansion of the input candidate features^8^. The NSH signal, which contains major power of NSH rhythm components inside a certain bandwidth, is reconstructed by the MVMD technique. However, power of the SH rhythm components has not considered properly.

Deep learning is the state-of-the-art technique, which consists of multiple layers to learn the noble features and then classify the input segments based on these LFs. The first research applying CNN for detection of SH/NSH rhythms produces the performance with Ac of 93.18%, Se of 95.32%, and Sp of 91.04%, which do not meet the AHA recommendations for the AED^14^. This maybe due to only a fCNN structure of 11 layer is considered with stand-alone ECG segment. Moreover, there is no attention to the CNN as the feature extractor, which can generate effective feature vector used as the input of the ML classifier.

**Figure 2:**
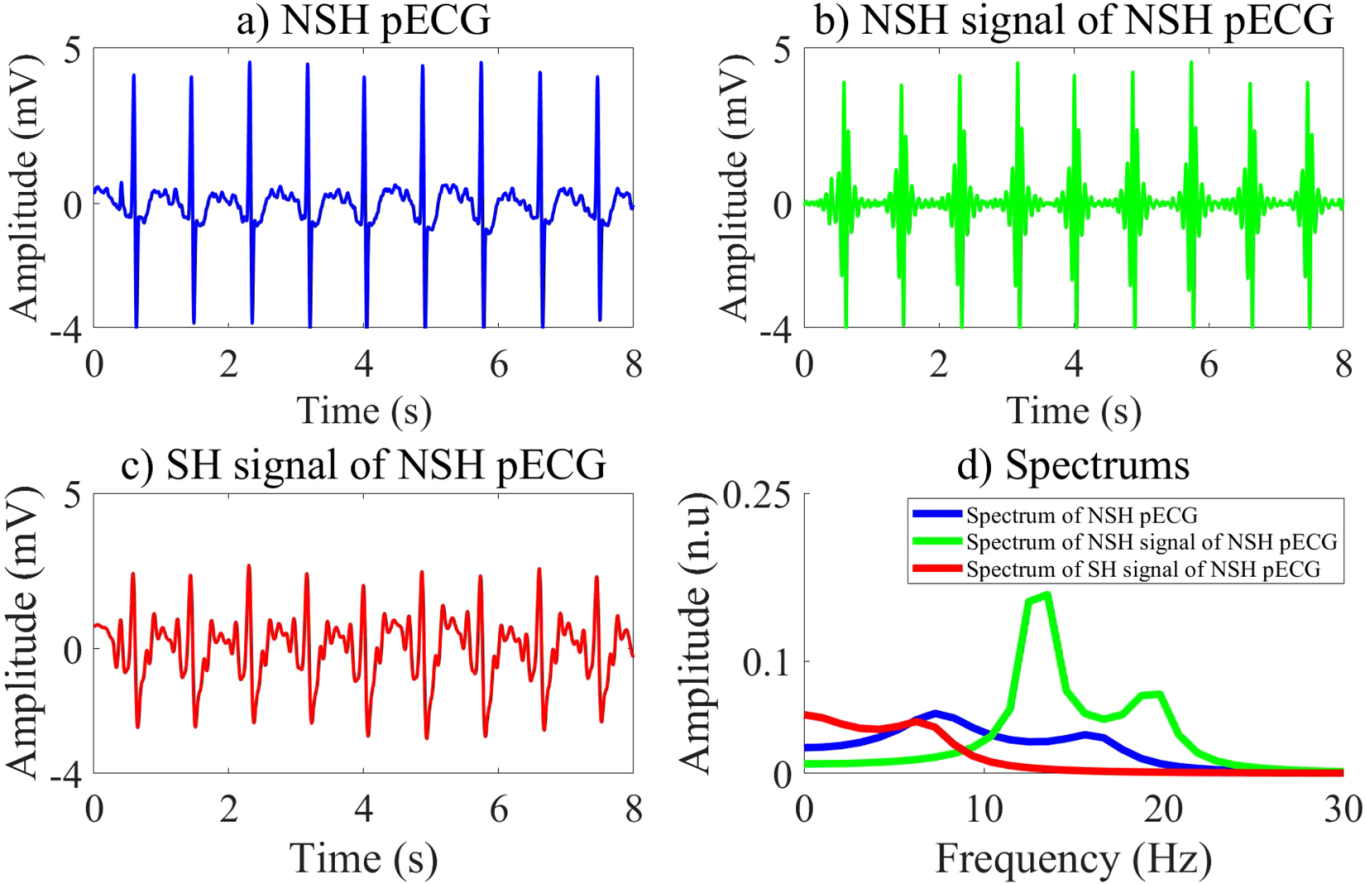
Input channels and their spectrums of the CNN for NSH pECG.

**Figure 3:**
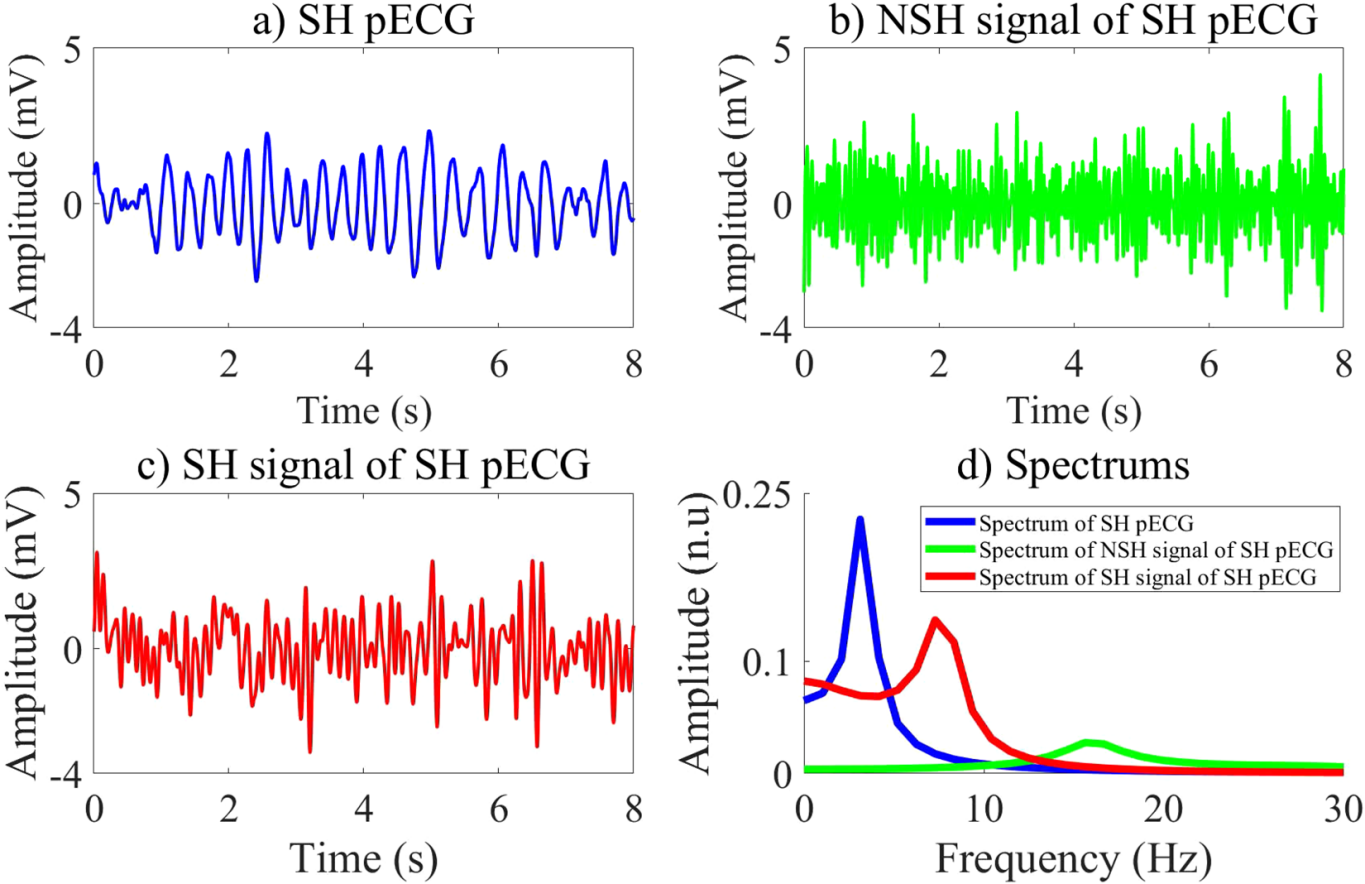
Input channels and their spectrums of the CNN for SH pECG.

In our research, we investigate different CNN structures by employing an exhaustive grid search with nested 5-folds CV to select the best values of the learning and structure parameters among conventional values. Moreover, the input of the CNN including three channels as shown in Figs 2 and 3, which are pECG, SH, and NSH signals reconstructed by the MVMD technique, certainly improves the quality of the LF vector. The performance results of 6 ML classifiers using the LF vectors are relative high and meet the AHA recommendations. The highest validation performance is archived for the BS classifier with Ac of 99.26%, Se of 97.07%, and Sp of 99.44% on 8 s-segment database. Here, the feature vector is extracted by the CNNE3 selected by the RF classifier on the training data. However, the validation performance of the fCNN does not meet the AHA recommendations for the AED with Sp of 89.25%, which is lower than recommended minimum value of 90%.

**Figure 4:**
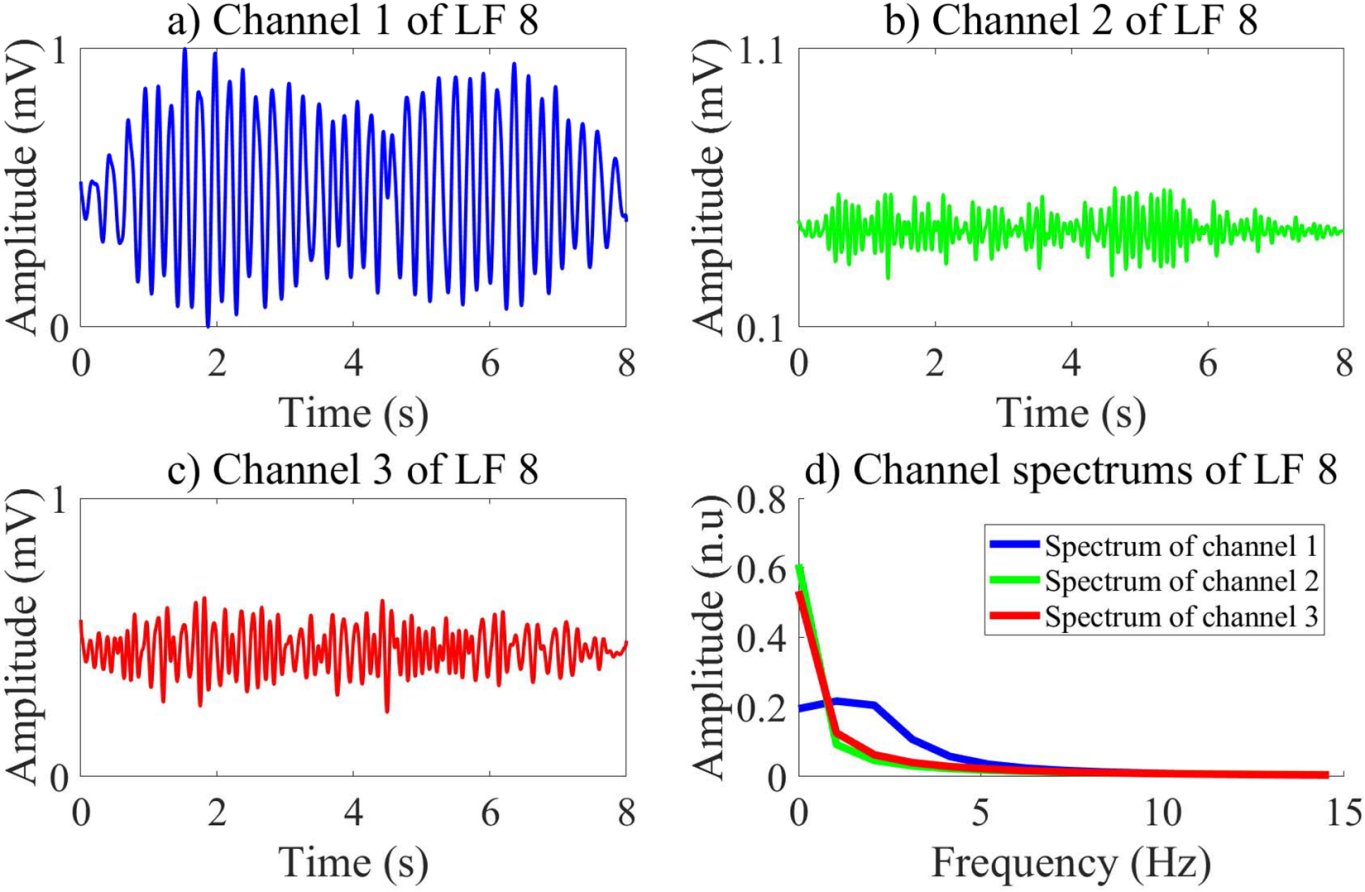
Channels and their spectrums of the LF 8.

**Figure 5:**
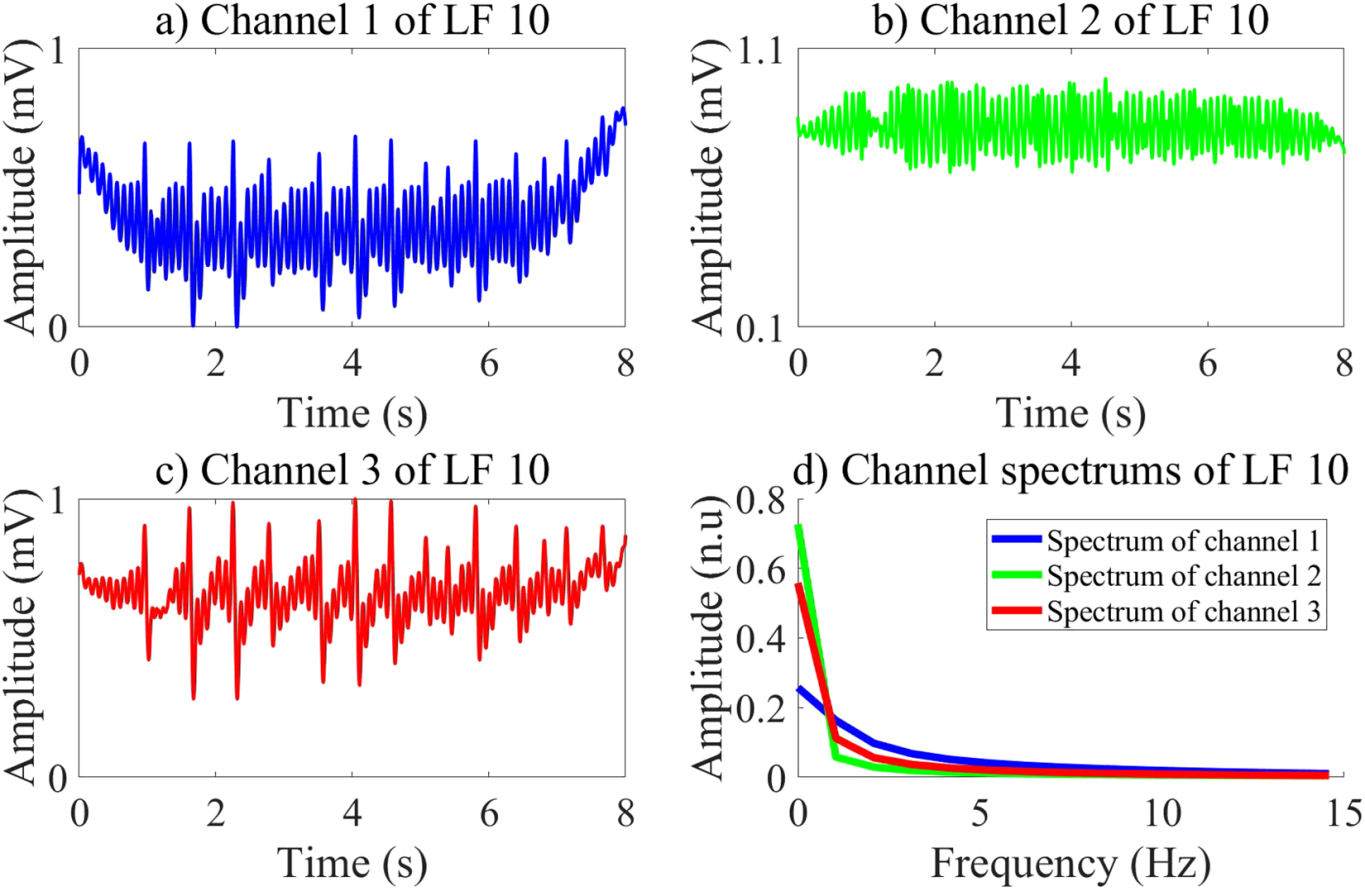
Channels and their spectrums of the LF 10.

The CNNEs learn various characteristics of input channels during training state. Each layer of the CNNEs can represent high level features, which then are used to activate the feature vector from the evaluation data. Here, the first FC is used to active a vector of 100 features corresponding to 100 outputs of this layer. Therefore, each LF of the pre-trained CNNEs on the first FC includes 3 channels. Totally, there are 300 channels, which can be divided into 2 groups in terms of channel waveforms and spectrums. The group 1 includes 49 LFs while 51 LFs are in group 2. Indeed, 2 groups of the LFs represent the binary classes of SH/NSH rhythms. Due to a larger number of channels, we visualize only 6 channels of the LF 8 and LF 10, which are typical representatives of 2 groups as shown in Figs 4 and 5. For the LF 10 in group 1 of Fig. 5, the channel 1 and channel 3 show the clear peaks, which maybe reflect the NSH pECG and SH signal as shown in Fig. 2. Moreover, there is no identified peak on all of 3 channels of the LF 8 in group 2 of Fig. 4, which maybe due to no peak on SH pECG, NSH, and SH signals as shown in Fig. 3. Another significant characteristic is that the amplitude of channel 2 is stronger for LF 10 than LF 8. This is because the channel 2 captures NSH components of input pECG. Therefore, the amplitude of this channel is higher for input NSH pECG and smaller for input SH pECG.

**Figure 6:**
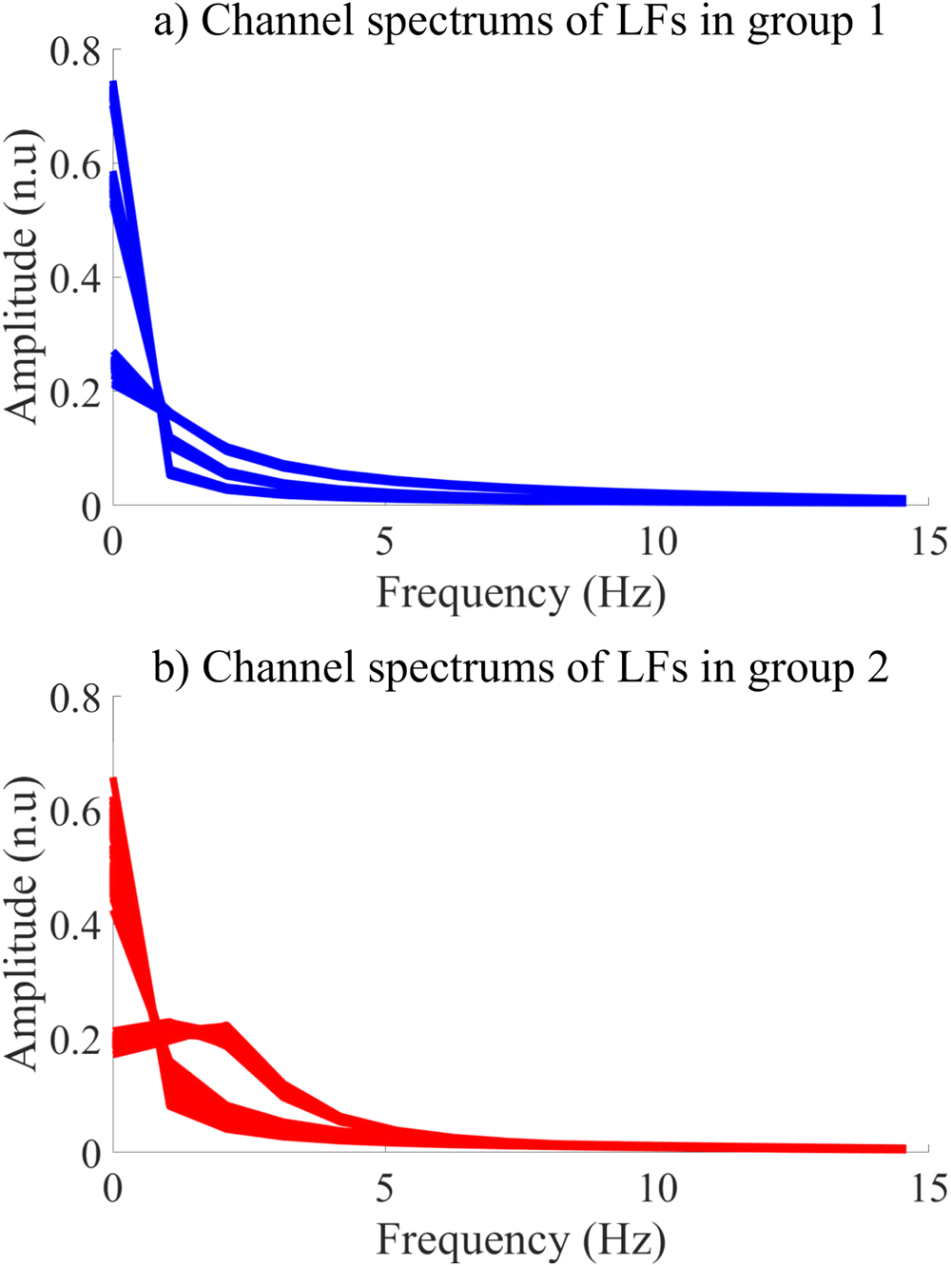
Spectrum of LF channels in 2 groups.

Generally, the channel power concentrates on a bandwidth from 0 to 3 Hz. The power of channel 1 is higher for the LF 8 than the LF 10. Moreover, both channel 2 and 3 show smaller power for the LF 8 than the LF 10. Indeed, the channel 2 of the LF 10 captures the NSH components of the input NSH pECG, which shows the largest power among the others. Furthermore, the SH pECG includes most of SH components regardless of the SH signal, which can be captured by the channel 1 of the LF 8. Therefore, the power of the channel 1 is emphasized for the input SH pECG. The Fig. 6 shows the spectrum of 147 channels of group 1 and 153 channels of group 2, respectively.

**Figure 7:**
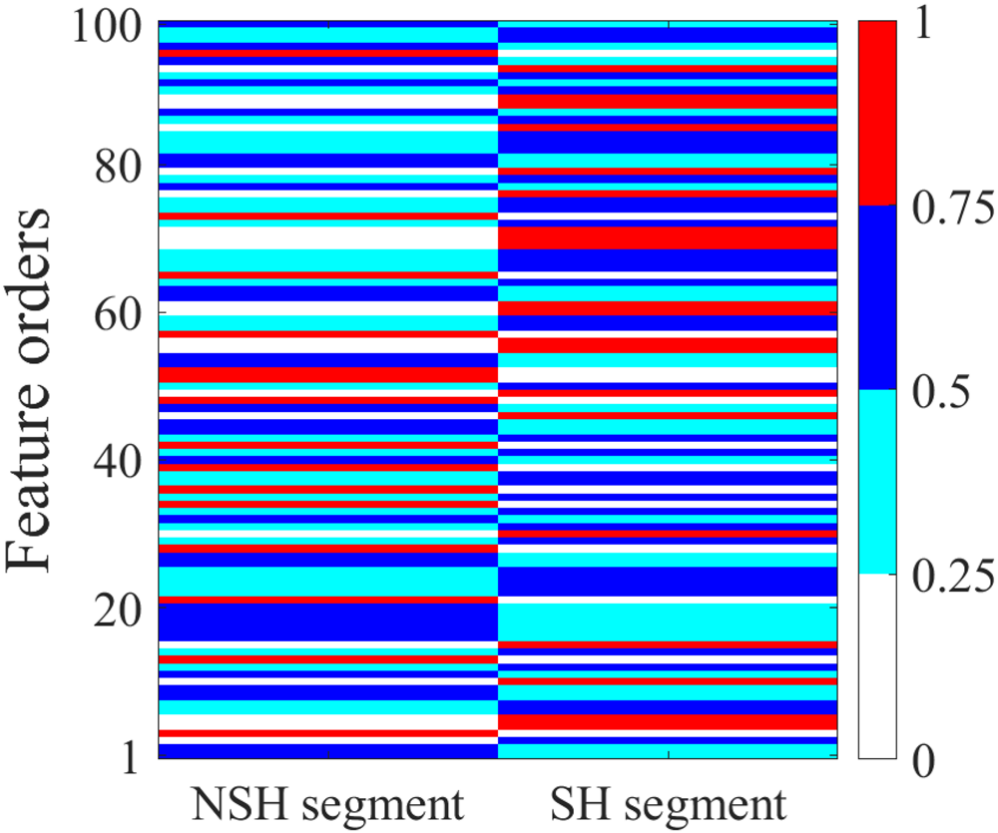
Visualization of feature vector for NSH and SH ECG segments.

The Fig. 7 shows the visualization of feature vector activated for NSH and SH pECG segments. The values of each features for the SH and NSH ECG segments are totally different, which makes the feature vector is extremely effective to classify the SH/NSH rhythms.

**Figure 8:**
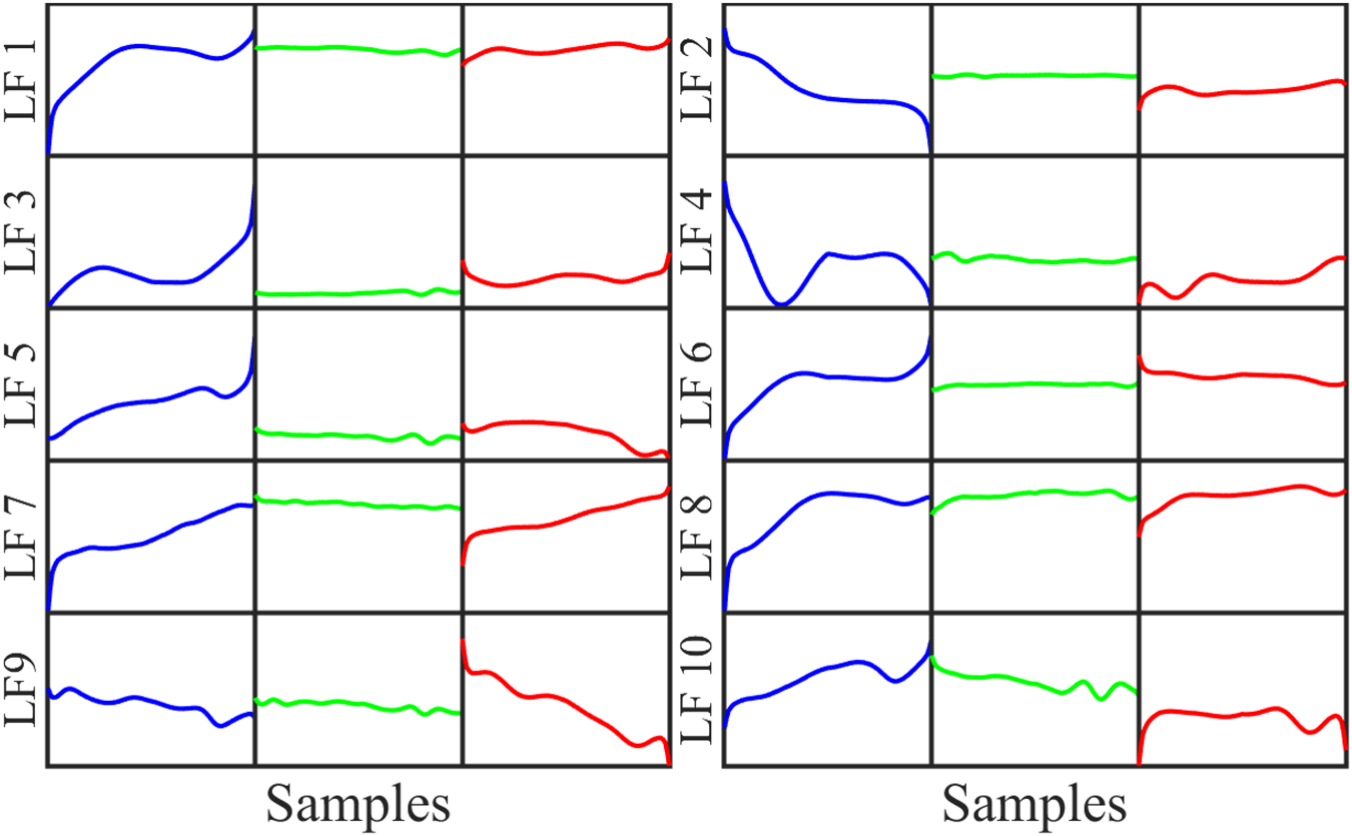
Visualization of 10 LFs on the first convolutional layer.

In Fig. 8, the waveforms of the LFs are different from each others and divided possibly into 5 groups. Moreover, the patterns of this LF are unorganized and not-useful apparently for further training or classifying SH/NSH rhythms.

**Figure 9:**
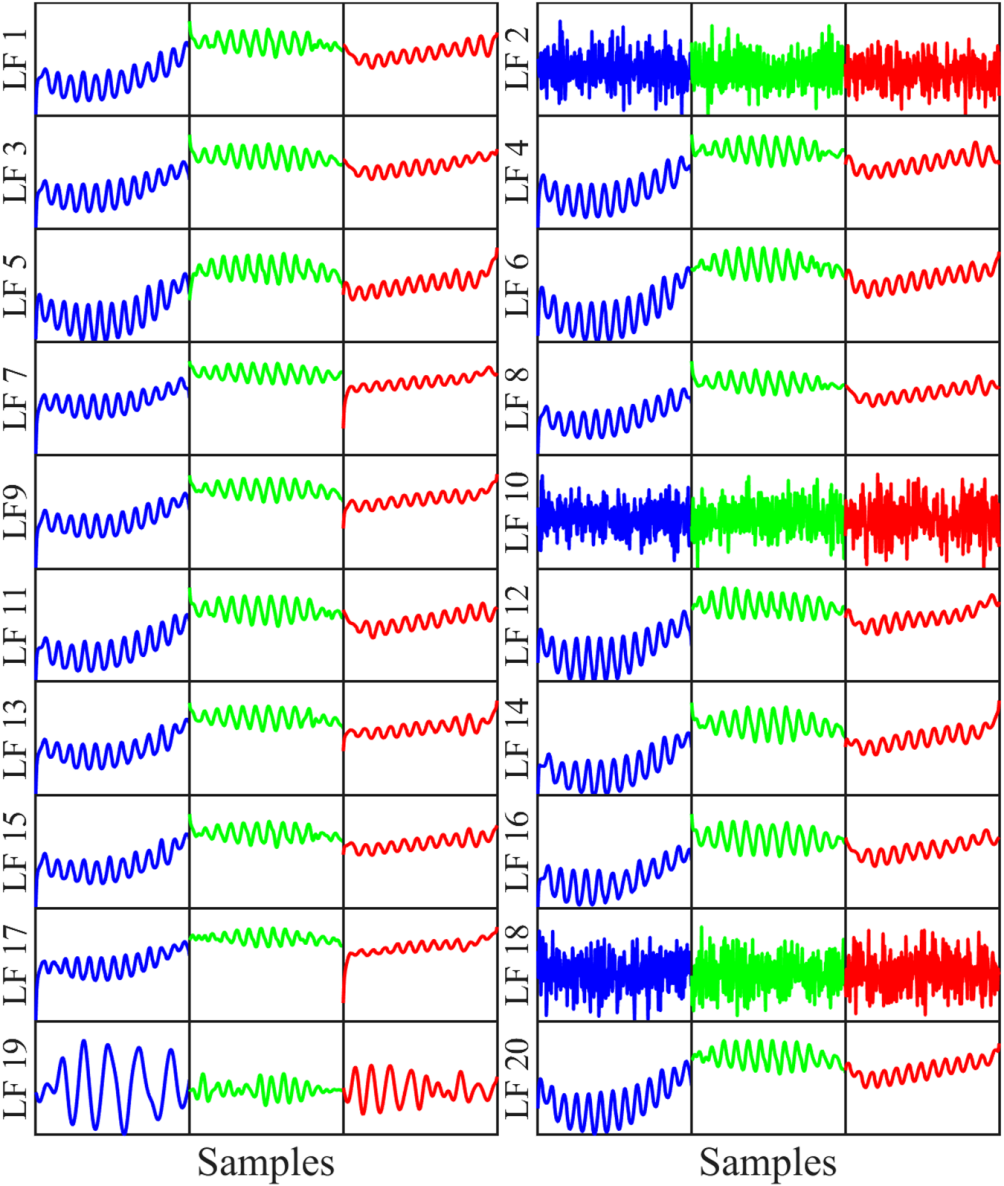
Visualization of 20 LFs on the second convolutional layer.

However, 19 LFs of the second convolutional layer can be separated clearly into 2 groups including 16 LFs and 3 LFs. One remaining LF, namely LF 19, which shows different waveforms from the others as shown in Fig. 9, is located as the third group.

**Figure 10:**
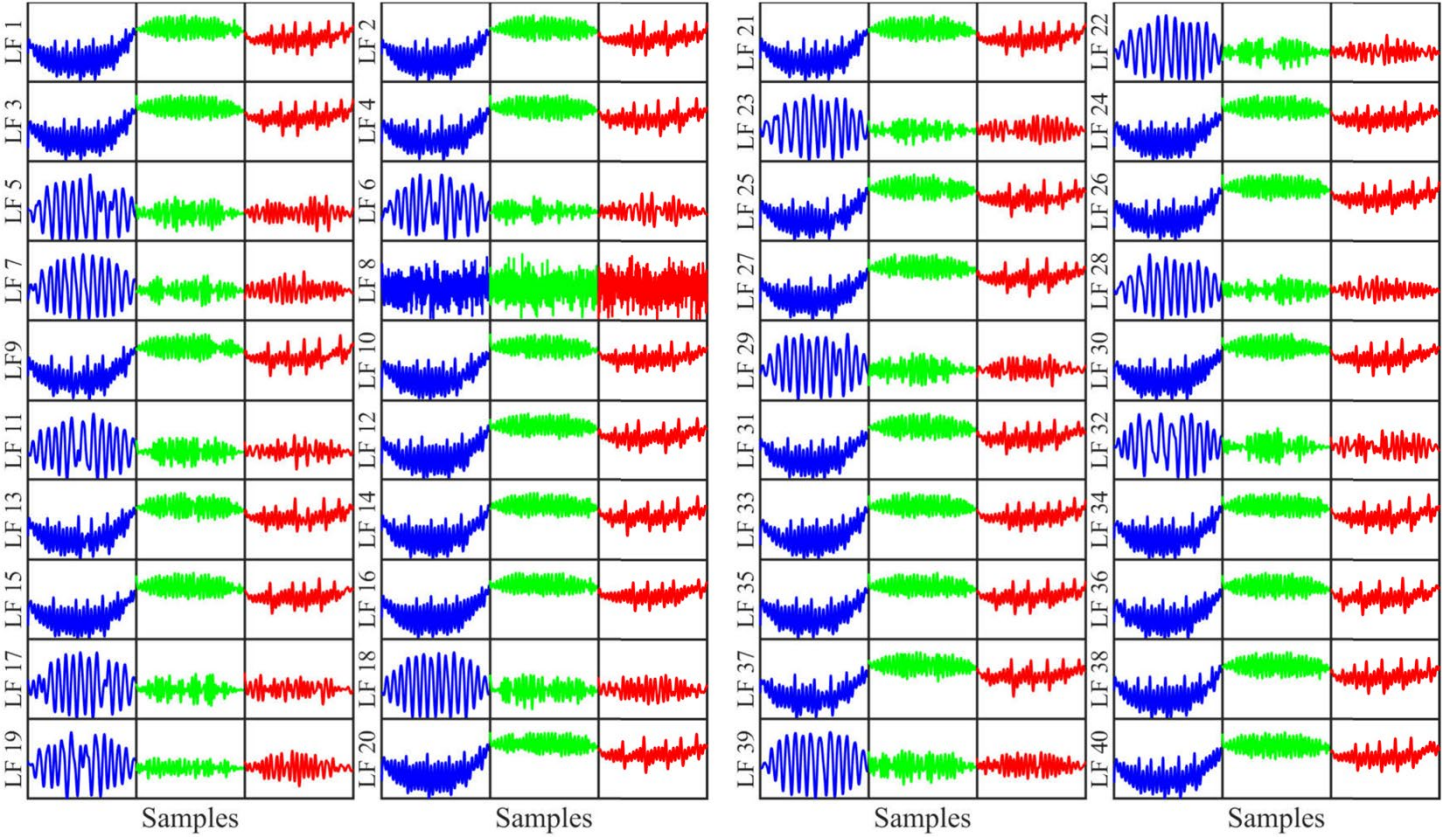
Visualization of 40 LFs on the last convolutional layer.

Similarly, 2 groups can be constructed from 39 LFs of the last convolutional layer due to 2 different categories of waveforms of these LFs. The waveform of the LF 8 is totally diferent from above LFs. Hence, this LF is contained in group 3 as shown in Fig. 10.

All the patterns in Figs 8, 9 and 10 are the production of the convolutional filters, which may not represent explicitly the characteristics and waveforms of the normal ECG or VF/VT rhythms. However, evolution of the LFs can be observed intuitively from the first to the last convolutional layer. Obviously, the LFs are more clearly fallen into small number of groups for the later than the former convolutional layer. Yet, minimum number of groups is 3 for second and last convolutional layers in comparison with 2 groups corresponding to 2 classes for the first FC layer. This shows the existence of redundant LFs extracted from convolutional layers, which are unfitted in classification or training a following ML classifier. It is noteworthy that the LF waveforms extracted from last convolutional layer in Fig. 10 are similar to those of the first FC as shown in Figs 4 and 5, while preceding convolutional layers release the LFs with significantly different waveforms. This implies that the LFs of the last convolutional layer carry more typical information of the input data because they learn and combine the characteristics of high level LFs of the preceding convolutional layers. In other words, the LFs are evolved during training process from the first to last convolutional layers. The evolved process is kept forward to the first FC layer, where more representative LFs are obtained obviously than that of last convolutional layer.

The rationale behind the use of first FC for the FE is that this layer determines the nonlinearity of high level features on last convolutional layer. This leads to increase in linearity of the feature vector activated on last FC in comparison with first FC. Consequently, the classification performance is reduced due to the decrease in nonlinearity of the feature vector. Indeed, the validated performance results of BS classifier using feature vector including 2 features extracted by the CNNE at the last FC are obtained with Ac of 95.65%, Se of 92.61%, and Sp of 96.46%. These values are clearly lower than those as shown in Table 2.

The validation performance of BS classifier using feature vector extracted by the CNNE is better than that of the fCNN as presented in Table 2. It is supposed that the fCNN uses the feature vector to classify directly the input segment at its output layer while the BS classifier learns the characteristics of input segment by using the feature vector. The secondary learning of the BS classifier definitely improves the performance in terms of SH/NSH rhythm detection.

A full 13-layer 1-dimension CNN using raw stand-alone ECG signal is proposed for atrial fibrillation detection^12^ due to this algorithm performance is better than that of the CNN using the conventional features extracted from stand-alone ECG signal. Therefore, the conventional features are no longer to consider in our work, where the CNN is used as the feature extractor, which shows a better classification performance than that of the fCNN. It is because the secondary learning of the BS classifier improve certainly the performance in terms of SH/NSH rhythm classification. Moreover, the MVMD technique is used to generate SH and NSH signal from ECG segment put into CNNE as three input channels, which improve definitely the quality of the LFs. An ensembled XGBOOST classifier and a feature set are suggested as the proposed algorithm^13^ in which the feature set consists of both conventional features extracted from stand-alone ECG segment and deep features constructed by the deep neural networks (DNN). However, the use of such feature combination makes it difficult to employ a FS algorithm, which is necessary for removal of irrelevant features and improvement of learning process. Furthermore, the conventional FE is time consuming, complexity and the utility of only fixed structure of the DNN for the FE leads to omit other efficient DNN structures. In our research, we pay attention to only deep features learned by the CNNE, which is simple and requires no conventional expertise-based FE and FS algorithms. Moreover, the quality of the LFs is improved by the use of pECG, shockability and nonshockability oriented signals as multiple input channels for the CNNE.

Table 4 compares the our proposed method with existing algorithms in terms of the SH/NSH rhythm classification performance. It is shown that the proposed method outperforms the existing methods in terms of classification performance. The method is also implemented for 5 s-segment data. The best validated Ac of 99.02%, Se of 95.21%, and Sp of 99.31% are produced by the SVM classifier using the feature vector extracted by the CNNE, which is selected by the SVM classifier on the 5 s-segment training data.

**Table 4:** Performance comparisons of the proposed method to existing algorithms.

Ref.	Appoaches	Type of method	Segment	Ac (%)	Se (%)	Sp (%)
Nguyen *et al*.^8^	- MVMD - GA, SFFS - SVM	ML	8 s	99.00	97.36	99.16
Acharya *et al*.^14^	- CNN	DL	2 s	93.18	95.32	91.04
Proposed SAA	- MVMD - CNNE - BS	ML and DL	8 s	99.26	97.07	99.44

## Conclusion

Correct detection of SCA is essential in terms of improving the chance of survival, especially for the OHCA situations, and reducing unnecessary defibrillation, which certainly causes the artificial SCA for the patients. Therefore, construction of the SAA design with high and reliable detection performance is the most important issue for the AED applications to ensure safe diagnosis.

In this work, we proposed a novel SAA applied for the AED using a combination of DL for FE and ML for classification. Here, three 1-dimension input channels are reconstructed by the MVMD and fed into the CNNE to improve the quality of the LFs. Moreover, the learning and structure parameters of the CNN are selected carefully by an exhaustive grid search with nested 5-folds CV. These deep features activated by the selected CNNE, which is less complexity and time consuming than conventional FE methods, are used to train the BS classifier. Obviously, the robust learning of the proposed SAA by using the LFs for the secondary training of the BS classifier improves definitely the final performance in terms of SCA detection. Indeed, the proposed SAA shows the validated performance results with Ac of 99.26%, Se of 97.07%, and Sp of 99.44% on the evaluation data, which are higher than that of existing SAAs using either fCNN or ML techniques.
